# Mixed results from a multiple regression analysis of supplemental instruction courses in introductory physics

**DOI:** 10.1371/journal.pone.0249086

**Published:** 2021-04-01

**Authors:** Eric Burkholder, Shima Salehi, Carl E. Wieman

**Affiliations:** 1 Department of Physics, Stanford University, Stanford, California, United States of America; 2 Graduate School of Education, Stanford University, Stanford, California, United States of America; National Taiwan University of Science and Technology, TAIWAN

## Abstract

Providing less prepared students with supplemental instruction (SI) in introductory STEM courses has long been used as a model in math, chemistry, and biology education to improve student performance, but this model has received little attention in physics education research. We analyzed the course performance of students enrolled in SI courses for introductory mechanics and electricity and magnetism (E&M) at Stanford University compared with those not enrolled in the SI courses over a two-year period. We calculated the benefit of the SI course using multiple linear regression to control for students’ level of high school physics and math preparation. We found that the SI course had a significant positive effect on student performance in E&M, but that an SI course with a nearly identical format had no effect on student performance in mechanics. We explored several different potential explanations for why this might be the case and were unable to find any that could explain this difference. This suggests that there are complexities in the design of SI courses that are not fully understood or captured by existing theories as to how they work.

## Introduction

Supplemental instruction (SI) is a commonly used approach in mathematics and chemistry education to provide additional help to students. The basic premise is that students who desire extra help or are determined to be at risk in introductory STEM courses will take an additional SI course to provide them with more targeted practice, individual attention, and, in some cases, social-psychological interventions. In some cases, SI resulted in improved grades for all students [1], and in other cases, disproportionately benefited underrepresented students [2]. SI courses taught by instructors or graduate TAs [3,4], as well as courses lead by undergraduate “peers” of enrolled students [5–10] have been shown to have a substantial impact in chemistry. Despite the popularity of SI in other disciplines, we could find no published studies in the physics education literature documenting the effects of SI courses, though there exist SI courses in physics and some more general studies suggesting positive benefits of such courses in physics [11,12]. We also note that SI courses are not always offered by the same department as the target course, but sometimes by centers of teaching and learning, the organizing college or school (e.g., the AEW program at Cornell University), or other campus groups.

Despite evidence of the positive effects of SI, the review by Dawson et al. found that SI is not consistently defined in the literature, and that most articles on the subject do not actually specify what happens in a particular session of the SI courses [13]. Rath et al. [14] provided the following description:

“Typical activities included guided discussions with extensive class participation (often following small group work), worksheets that were completed both individually and in groups, peer instruction, preparation of study resources, kinesthetic and visual modeling of problems, practice tests, and trivia-style games. Particular emphasis was placed on the concepts, content, and vocabulary from the lecture, but before lab exams some time was spent reviewing methods, data analysis, and the interpretation and principles underlying observed outcomes of various laboratory experiments”.

However, Dawson et al. note that such descriptions, when present, are rarely supported by observations that support these descriptions [13]. They also find large variations in the number of participants in a particular session, and what constitutes sufficient attendance to qualify as an SI participant. Dawson et al. say that the role of the leader of a SI course session (often a successful undergraduate student, but sometimes an instructor or graduate student) is “facilitating discussion around course content, and related study skills, and for preparing learning activities such as worksheets, group work, problem-solving exercises, or mock exams for their students.” They also say that students in attendance are responsible for “teaching each other the course content and for working together to solve problems.” Despite variations in SI course design, they often share a common element: cooperative group problem-solving [15–17]. In this model, students work together in small groups on relevant problems while the instructors circulate the room to monitor discussion and provide targeted feedback to the groups. This intervention is thought to be effective because it encourages students to better monitor and be more aware of their own learning [18–20].

The effectiveness of SI courses is most often measured by grades in the target course (the course which the SI course is accompanying). Typically, researchers will use a quasi-experimental design and compare the course grades of students in the SI course with students not enrolled in the SI course, but they do not randomly assign students to these groups. They will then use *t*-tests to determine if the difference is significant, though Dawson et al. report that few studies provide effect sizes (e.g., Cohen’s *d*) [13]. However, this literature on SI is likely generally biased to only report instances in which SI courses were beneficial, so it is difficult to say how helpful these interventions are across all iterations. Another common problem in the literature on SI courses is the issue of self-selection bias. Even if there seems to be a positive effect of the SI course when examining final exam grades, it is difficult to disentangle the effects of the intervention from student characteristics that may have made students more likely to enroll in the SI course, which are nearly always optional. Indeed, some research has shown that providing additional benefits to students does not work as intended, because the students most at risk are less likely to use those resources [21]. Researchers have used measures of prior preparation (e.g., SAT/ACT scores, GPA) and measures of motivation as control variables to try and address the non-random nature of participation in SI courses. Some studies still find effects of SI using these controls (E.g., Ref. [22]). One study [23] suggests that the effects of SI courses are greater when attendance is mandatory, but that motivation is lower among students for whom the SI courses are mandatory. This suggests that, while motivation plays some role in the positive effects of SI courses, it is not the whole story.

Stanich et al. addressed the issue of self-selection in their work [9]. They recruited SI course participants by emailing students who scored in the bottom quartile of the chemistry placement exam and working with the Office of Minority Affairs and Diversity at their university. More students volunteered for the SI course than they were able to accept into the program, so they had a natural control group. Students were randomly selected to participate in the program from the list of volunteers. In that work, their SI course, which included cooperative group problem-solving, study skills development, and social-psychological writing interventions, showed a substantial positive effect. SI course participants scored much better than those who volunteered for the course but were not accepted, and they scored the same as the other students in the class who scored in the top three quartiles on the placement exam.

Stanford University has long used SI courses in introductory chemistry and math courses. More recently, the physics department implemented SI courses for physics 1 and physics 2 –the introductory calculus-based mechanics and electricity and magnetism (E&M) courses for scientists and engineers; these supplemental courses were called Phys 1A and Phys 2A. Student feedback on these courses was very positive, but there was no analysis of whether these SI courses had a positive impact on student course performance (i.e., grades in the course or on exams). To this end, we conducted a quantitative study of Phys 1A and 2A to determine if these courses were helping the students the courses were created to help—less prepared students. We posed the following research question:

1. Do Phys 1A and 2A have a positive effect on students’ final exam grades in Phys 1 and 2, and do these courses disproportionately benefit less prepared students?

To answer this question, we use multiple linear regression to predict final exam grades as a function of high school physics preparation (measured by concept inventory scores, SAT/ACT math scores, and prior math coursework) and participation in Phys 1A or 2A. We use final exam score rather than course grade because it is a comprehensive measure of content knowledge covered in the course that is the primary determinant of the students’ grades in these courses, and in prior work we have found that it is a more linear and consistent measure of performance than the course grades. The latter tend to be a very non-normal distributions, compressed to the top of the scale in a nonlinear way. As a result, we find that linear regression models explain much less of the variance in course grades than they do in final exam grades. In the next section, we provide a detailed description of Phys 1A and 2A. We then present our quantitative analysis and discuss the results.

## Course descriptions

### Phys 1 & 2

The structures of Phys 1 and Phys 2 were nearly identical. Both courses had three 50-minute lectures a week and an 80-minute discussion section once a week that was led by a teaching assistant. The lectures made limited use of clicker questions. In the discussion sections, students would solve problems, often adaptations from Tutorials in Introductory Physics, but there was no formal group problem-solving activity. Both courses had two midterm exams and a final exam which constituted approximately 80% of the final course grade. The remainder of the grade, which had little variation, consisted of grades from weekly problem-sets (which included problems from Mastering Physics and typically required several hours each week) and in-class participation (measured by answers to clicker questions). Phys 1 covered kinematics and projectile motion, forces and static equilibrium, uniform circular motion, conservation of energy, conservation of momentum, and torque and conservation of angular momentum. Phys 2 covered Coulomb’s law and electrostatics, Gauss’ law, capacitance and dielectrics, simple circuits, Ampere’s law, the Biot-Savart law, Faraday’s law, Lenz’s law, and Maxwell’s equations. Phys 1 used the textbook by Young and Freedman [24], while Phys 2 used that by Knight [25]. Both courses also had optional labs, which were separate courses taken by approximately half the students in Phys 1 and 2. The enrollments for each course in 2017 and 2018 are given in Table 1.

**Table 1:** Enrollment numbers for Phys 1(A) and Phys 2(A).

Course	2017 Enrollment	2018 Enrollment
**Phys 1**	463	518
**Phys 1A**	120	79
**Phys 2**	422	460
**Phys 2A**	22 (21 also in Phys 1A)	44 (23 also in Phys 1A)

### Phys 1A

Phys 1A was a one-unit course which met for 110 minutes (with typically a 10-minute break in the middle) once per week in addition to the regular Phys 1 discussion sections. Thus, it was typically 100 minutes per week of supplemental instruction, on top of the regular 230 minutes per week of instructional time students had in Phys 1 and Phys 2. Originally, this course was optional and there was no screening of students who wanted to enroll based on need. Over a period of several years, we found that students in Phys 1A consistently rated the course highly, but on the final exam they consistently scored about 0.5 standard deviations lower than the other students in Phys 1, on average. There were several changes in the course over the years in an unsuccessful attempt to reduce this difference. Initially, the course focused on reviewing lecture material. Then it was changed to small group problem-solving. Eventually, an application and screening process was implemented, and the course started to focus more on fundamental ideas and problem solving. The 0.5 standard deviation difference remained unchanged throughout these changes.

In 2017 and 2018, students had to complete an application and be approved by the instructor to enroll in Phys 1A. Applicants were screened to give preference to students with less physics and math preparation. Most of the class time was spent with students working in small groups of 3–6 on problems, while the instructor and TAs circulated throughout the room to monitor discussion and answer questions. These problems were written by the course instructors and were designed to cover the ideas that were most difficult for students (e.g., adding vectors, identifying relevant forces, etc.), and to teach elements of good problem-solving practice [26]. The instructor would call students back from their small groups to review the solutions to the problems with the whole class at specified intervals. In addition to this small group work, students would practice timed exam problems so that they could get used to high-stakes time-constrained problem-solving. An example course worksheet may be found in the Supplemental Material. In 2017 we conducted a detailed analysis of performance in Phys 1A similar to the analyses we present below. We found no effect of Phys 1A in 2017 on course or exam performance, and thus reformed the course again for 2018. S. S. and C. E. W. designed a template to help students learn good problem-solving practices, based on prior research [26]. The template asked students to explicitly engage in steps of problem-solving such as planning their approach, listing the assumptions they were making, and reflecting on their solution [27]. For all iterations of both Phys 1A and 2A, the TA to student ratio was large (and similar for both courses), and the TAs were specially selected on the basis of having previously shown themselves to be particularly good at working with struggling students.

### Phys 2A

Phys 2A was very similar to Phys 1A. It was a one-unit course which met for 110 minutes (with 10-minute break) once per week in addition to the regular Phys 2 discussion sections. Enrollment was open in 2017, but in 2018 students had to complete an application to enroll in Phys 2A. Prior enrollment in Phys 1A did not guarantee a student a spot in Phys 2A. Like Phys 1A, the majority of class time was spent with students working in groups of 3–4 on problems while the instructor and TAs circulated the room to monitor discussion and answer questions. The class sessions would begin with a recapitulation of ideas covered in lecture that week, with particular focus on ideas that students struggled with, similar to the early iterations of Phys 1A. Students would be asked to discuss relevant ideas in small groups and come up with mathematical and conceptual definitions of different ideas. The instructor would then ask groups of students to share their definitions with the whole class to develop the ideas fully for the whole class. Students would then begin working in their small groups on problems. The problems were either examples used in other parts of the Phys 2 course or from the textbook [25]. For an example worksheet from Phys 2A, see the Supplemental Material. The worksheets would contain problems that reviewed materials already covered in lecture or prepared students for upcoming lectures. The instructor would call students back from their small groups to review the solutions to the problems with the whole class. After midterm exams, the instructor would sometimes review answers to the free-response questions with students to make sure they understood the problems. All of these were similar to the conduct of 1A, except for more care in the creation of the 1A problems in the later iterations, to try to better target specific areas of student difficulty.

Phys 1A and 2A had applications required for enrollment, so the prior literature suggests that these students are more motivated than students who do not enroll in the SI courses, but have similar levels of high school physics and math preparation. Furthermore, grades in the SI courses were based only on attendance, ensuring that attendance was high.

## Methods

We collected data on students’ incoming physics and math preparation, as well as their physics 1 & 2 course performance (as measured by final exam grades), to determine whether Phys 1A and 2A had any effect on course performance after controlling for student prior preparation. For Phys 1A, we collected students’ FMCE pre-scores and SAT/ACT math scores, as we had previously found those two variables were the only predictors of Phys 1 course performance [28]. For Phys 2A, we collected FMCE pre-scores, SAT/ACT math scores, CSEM pre-scores, Phys 1 final exam scores, and whether a student had already taken vector calculus prior to Phys 2A. We found these variables to be predictors of performance in Phys 2 in previous work [29]. The FMCE is a short conceptual test of mechanics and motion commonly used in physics education research, and the CSEM is a short conceptual test of basic electricity and magnetism concepts. Students provided written consent for the use of anonymized course data in future research at the beginning of each course when taking the FMCE or CSEM. Students who did not give consent were removed from analysis. This work was determined exempt from review under Stanford University protocol IRB-48006.

We then ran multivariable regression analysis for both 2017 and 2018 to predict Phys 1 and Phys 2 final exam scores as a function of incoming preparation and participation in Phys 1A or Phys 2A, and the interaction of incoming preparation and participation in 1A or 2A, respectively. We scaled final exam scores and measures of incoming preparation such that the regression coefficients as shown below would be in units of standard deviations. We used multiple imputation with predictive mean matching to account for missing data. We imputed 20 different data sets, and then pooled the results of the regression models of all 20 data sets using the mice package in R.

Multiple imputation is an alternative to complete case analysis—simply deleting participants for whom complete data is not available. Complete case analysis is known to introduce biased errors of parameters [30]. Multiple imputation is an appropriate solution to this problem when data are missing at random—i.e. when the probability of data being missing is dependent on other observed variables, but not on the missing values themselves. For example, we are missing some FMCE pre-scores. It is possible that missing the first day of class is more likely for students who are less likely to perform well on the final exam. Thus, the missingness of the FMCE score is explained by the final exam score and not dependent solely on the FMCE score itself. Thus multiple imputation can account for these differences. For more detail on multiple imputation, see [30].

## Results

The results from the analysis of Phys 1 final exam scores are in Table 2. In 2017 model a, we calculate whether there was an overall effect of Phys 1A enrollment after controlling for students’ prior preparation. In 2017 model b, we add an additional term to model a to see if the effect of Phys 1A enrollment is different for students with different levels of prior preparation. 2018 model a and 2018 model b are the same, except with the population of students from 2018. As in previous work, we found that FMCE pre-score, and SAT/ACT math score are strong predictors of course performance. We also tested for the differences associated with taking different levels of calculus courses and found no effect. However, controlling for these measures of incoming preparation, we found no statistically significant main effect or interactive effect of taking Phys 1A. This indicates that two students with the same scores on these measures of incoming physics and math preparation, one enrolled in Phys 1A and the other not, will receive the same final exam score in Phys 1. The lack of main effect for Phys 1A, as shown by the insignificant coefficient of Phys 1A in row 4 of Table 2, shows that the SI course has not improved the performance in Phys 1 of students who took it. The lack of an interactive effect, as suggested by insignificant coefficient of Phys 1A x SAT/FMCE (rows 5 and 6, Table 2), shows that taking this SI course did not moderate the effect of incoming preparation on Phys 1 final exam performance, and thus, this SI was not effective in addressing the impact of differences in prior preparation on performance in Phys 1. This apparent lack of benefit is in notable contrast to the student evaluations of the 1A course, which were overwhelmingly positive, many making comments such as “I would never have survived physics 1 without 1A!”.

**Table 2:** Regression models for Phys 1 final exam performance as a function of incoming preparation and enrollment in Phys 1A. Coefficients are in units of standard deviations and the numbers in parentheses are the standard errors.

Phys 1 Final Exam	2017 Model a	2017 Model b	2018 Model a	2018 Model b
FMCE Pre-Score	0.44*** (0.043)	0.45*** (0.045)	0.35*** (0.045)	0.35*** (0.048)
SAT/ACT Math Score	0.22*** (0.043)	0.26*** (0.053)	0.32*** (0.044)	0.33*** (0.053)
Phys 1A	-0.021 (0.070)	-0.082 (0.090)	0.059 (0.11)	-0.042 (0.13)
Phys 1A x SAT		-0.055 (0.048)		-0.038 (0.098)
Phys 1A x FMCE		-0.053 (0.092)		-0.14 (0.14)
R-squared	0.30	0.31	0.31	0.32

*** p < 0.001,

** p < 0.01, * p < 0.05, † p < 0.10. 2017 models and b include all students enrolled in Phys 1 in 2017 (N = 463). 2018 models a and b include all students enrolled in Phys 1 in 2018 (N = 518).

The results from the similar analysis of Phys 2 final exam scores are in Table 3. In 2017 model a, we calculate whether there was an overall effect of Phys 2A enrollment after controlling for students’ prior preparation. In 2017 model b, we add an additional term to model a to see if the effect of Phys 2A enrollment is different for students with different levels of prior preparation. 2018 model a and 2018 model b are the same, except with the population of students from 2018. Adding the interaction term between incoming preparation and enrolling in SI did not improve the model fit for either year (as suggested by the same R^2^ of model a and model b), and neither of the interaction terms was significant at the P = 0.05 level. Therefore, we take 2017 Model a and 2018 Model a to be the simplest, best-fitting models for interpreting SI course effects. As in previous work, we find that SAT/ACT math score, CSEM pre-score, and prior Vector Calculus experience are all significant predictors of performance in Phys 2. In 2018, FMCE is also a significant predictor, stronger than in 2017, likely due to differences in the respective Phys 2 final exams. We find an inconsistent effect of Phys 2A. In 2017, for two students with the same FMCE, CSEM, and math SAT scores, one enrolled in 2A and one not, the student enrolled in 2A performed 0.79 (0.19) standard deviations better on the final exam (2017 model a row 6), which is a large effect size. In 2018, the effect size was 0.13 (0.14) standard deviations and was not statistically significant (Model 2018 a). In both 2017 and 2018, we found no significant interaction effects between incoming preparation and enrolling in SI course, suggesting that if Phys 2A is effective, it is equally effective for all students.

**Table 3:** Regression models for Phys 2 final exam performance as a function of incoming preparation and enrollment in Phys 2A. Coefficients are in units of standard deviations and the numbers in parentheses are the standard errors.

Phys 2 Final Exam	2017 Model a	2017 Model b	2018 Model a	2018 Model b
FMCE Pre-Score	0.14† (0.070)	0.14† (0.070)	0.22*** (0.061)	0.22** (0.065)
SAT/ACT Math Score	0.23** (0.069)	0.25** (0.080)	0.23*** (0.054)	0.24*** (0.062)
CSEM Pre-Score	0.35*** (0.055)	0.33*** (0.055)	0.23*** (0.046)	0.25*** (0.060)
Prior Vector Calculus	0.44** (0.11)	0.44*** (0.12)	0.24* (0.099)	0.24* (0.11)
Phys 2A	0.79*** (0.19)	0.56 (0.56)	0.13 (0.14)	-0.31 (0.36)
Phys 2A x FMCE		-0.25 (0.29)		0.0028 (0.23)
Phys 2A x SAT		-0.11 (0.13)		-0.072 (0.14)
Phys 2A x CSEM		0.092 (0.42)		-0.43 (0.27)
Phys 2A x Vector		0.040 (0.47)		0.15 (0.33)
R-squared	0.33	0.33	0.32	0.32

*** p < 0.001,

** p < 0.01,

* p < 0.05,

^†^ p < 0.10 2017 models and b include all students enrolled in Phys 2 in 2017 (N = 422). 2018 models a and b include all students enrolled in Phys 2 in 2018 (N = 460).

A different perspective is provided by adding to the model the students’ Phys 1 final exam score as shown in Table 4. When this is included in the model, not surprisingly, the other measures of prior preparation are less important. This score is a very strong predictor of Phys 2 final exam grade and including it in the model explains far more variance in final exam scores than the models in Table 3. This is not surprising as the Phys 1 final exam score measures skills related to performance in physics beyond those measured by the concept inventories and SAT/ACT math scores—e.g., study skills, problem-solving, psychological adjustments to university physics, instructor expectations in this department, etc. These are more complete measures of preparation for performance in Phys 2.

**Table 4:** Regression models for Phys 2 final exam performance including Phys 1 final exam as a predictor. Coefficients are in units of standard deviations and the numbers in parentheses are the standard errors.

Phys 2 Final Exam	2017 Model a	2017 Model b	2018 Model a	2018 Model b
FMCE Pre-Score	-0.015 (0.062)	-0.018 (0.063)	0.047 (0.054)	0.043 (0.051)
SAT/ACT Math Score	0.11 (0.069)	0.12† (0.070)	0.081 (0.046)	0.076 (0.047)
CSEM Pre-Score	0.16** (0.048)	0.16** (0.049)	0.034 (0.047)	0.041 (0.047)
Prior Vector Calculus	0.29** (0.10)	0.29** (0.10)	0.12 (0.074)	0.12 (0.080)
Phys 1 Final Exam	0.55*** (0.060)	0.56*** (0.061)	0.71*** (0.041)	0.71*** (0.043)
Phys 2A	0.63*** (0.17)	0.54** (0.17)	0.30** (0.11)	0.30* (0.14)
Phys 2A x Phys 1		-0.36* (0.18)		0.014 (0.11)
R-squared	0.51	0.52	0.62	0.62

*** p < 0.001,

** p < 0.01,

* p < 0.05,

^†^ p < 0.10 2017 models and b include all students enrolled in Phys 2 in 2017 (N = 422). 2018 models a and b include all students enrolled in Phys 2 in 2018 (N = 460).

Less obvious, when Phys 1 exam score is added to the model, the impact of 2A became larger and statistically significant for 2018, as did the 2017 Phys 2A x Phys 1 final exam interaction term. As can be seen in, the interaction effects show that Phys 2A had a greater benefit for those who performed *poorly* in Phys 1 in 2017 (as indicated by the negative sign on the coefficient, which when multiplied by a below average, or negative, z-score becomes a positive effect), and it provided a significant benefit for everyone in 2018, regardless of their Phys1 final exam grades, although a smaller benefit than in 2017. This result is encouraging, because it suggests that Phys 2A is making Phys 2 more equitable for students who were less successful in Phys 1. The reason that Phys 2A is significant in the 2018 model in Table 4 but not Table 3 is not clear but is related to the correlation between the different variables in the regression models. In the discussion below, we will use the results in Table 4 to interpret the effectiveness of Phys 2A, as it is a better model, in that it explains more variance of Phys 2 final exam score (compare R^2^ in Table 4 with R^2^ in Table 3).

## Discussion

The most notable result is that the two SI courses, despite very similar structures and approaches, varied greatly in their effectiveness. Phys 1A is never effective across many years, enrollments, and instructional approaches. In contrast, Phys 2A appears to be effective, although with different results for different years. We have seen a fairly similar analysis of the impact of the SI course in the first term general chemistry course at Stanford, and although the data was not as detailed as what we present here, the lack of measurable benefit was the same as we see here with Phys 1A. This indicates that the mechanisms behind SI course interventions need to be better understood before such interventions are widely adopted. In the following section, we examine three possible reasons why Phys 1A was ineffective while Phys 2A was effective: differences in the student populations, differences in students’ abilities to navigate college courses, and differences in course structure. We then discuss possible reasons for the difference in the effectiveness of Phys 2A between 2017 and 2018.

### Possible reasons for differences in benefit between Phys 1A and Phys 2A

#### Differences in student populations

Phys 1 was 55% first-year students, while Phys 2 was only 38% first-year students. Many students choose to wait a year between taking Phys 1 and Phys 2, because Phys 1 is a prerequisite for many introductory engineering courses, while Phys 2 is not. Could it be that the SI course is effective for more experienced students and not for the first-year students, and as there were more first year students Phys 1A, the course was not effective? We tested this by running the regression models above separately on the students who are and are not first-year students. We saw the same results. Phys 1A had no effect for first-year or beyond-first-year students, and Phys 2A had an effect for both first year and beyond-first-year students in 2017, thus ruling out this potential explanation.

#### Differences in students’ abilities to navigate college courses

Another possible explanation for the difference between Phys 1A and Phys 2A is that students in Phys 2A have now taken a physics course and better understand how university physics courses are structured and how to best navigate them. Could the additional instructional time measurably help the 2A students, because things like time management and test-taking issues are not major factors for students in Phys 2 but were in Phys 1? We tested this by seeing if Phys 2A had a larger effect for students who were more successful in Phys 1. The negative sign on the significant interaction effect in Phys 2 2017 showed just the opposite effect, while for 2018 the interaction term was not significant (row 8 Table 4). This indicates this explanation is unlikely.

#### Differences in course structure/instruction

Another possible explanation for the difference between the effects of Phys 1A and Phys 2A is that they were taught differently. The instructors in Phys 2A might simply have been more effective than those in Phys 1A. This also seems unlikely. As noted above, the basic structure and methods of both SI courses were essentially identical. In addition, most of the instructors involved in teaching both of the courses had many years of experience teaching using active learning methods, and one of the instructors for Phys 2A in 2017 was also an instructor for Phys 1A in 2018. The other instructor of Phys 1A in 2018 was C. E. W., who is highly experienced in active learning and had provided guidance as to how to teach both 1A and 2 A in previous years. The consistent performance gap between Phys 1A students and Phys 1 students not-in-1A through multiple years of different instructors and different instructional foci also suggests there are more fundamental reasons for its lack of impact. There is some possibility that an instructor effect contributed to the differences in impact between the two years in 2A, as discussed below.

Another possible reason for the 1A-2A difference might be the exam format. The Phys 2 exams were 40% multiple choice questions which test students’ memorization of important facts and concepts from E&M, while the Phys 1 exams were entirely free-response questions. Perhaps Phys 2A was only successful because they reinforce the concepts needed to succeed on the multiple-choice section? We were able to rule out this explanation by running the models from Table 4 using the scores from multiple choice and free response questions as separate outcome variables. In 2017, we found a slightly larger effect of Phys 2A on the multiple-choice section (0.70 standard deviations), but the effect on free-response questions was still large (0.50 standard deviations). So, this difference in exam format does not explain the difference between in the impacts of the two SI courses.

A final potential explanation concerns the range of student preparation in the two courses relative to the material covered. Students in Phys 1 had a very wide range of relevant preparation. Some students had little or no high school physics preparation, while many others had taken good AP physics courses covering essentially all the material in Phys 1. Comparatively, in Phys 2, few students had any experience with E&M content, so they all had approximately the same level of prior preparation in that material. Thus, the average Phys 1A student starts much farther behind the average Phys 1 student with regard to knowing the material covered in the course than is the case with Phys 2A and Phys 2 students. This can be seen in Table 5. The average differences between the Phys 1 and Phys 1A students’ scores on the FMCE, as well as the standard deviations for Phys 1A students’ FMCE score, are substantially higher than the corresponding values for Phys 2 on the CSEM. We hypothesize that a 100 minute per week intervention is simply insufficient to make a measurable impact on final exam grades for the Phys 1A students, because the incoming preparation gap is so large, and the typical Phys 1 students are already having 230 minutes of instructional time plus spending another 200–300 minutes per week studying the material and doing homework. In principle, we would expect that if this explanation were correct, it would show up as an interaction term between preparation and taking Phys 1A. However, if the gap in preparation was too large that interaction effect would also be insignificant. This remains the explanation we believe is most likely.

**Table 5:** Mean and standard deviation of FMCE scores for Phys 1 and Phys 1A, and CSEM pre-scores for Phys 2 and Phys 2A.

	2017	2018
Phys 1A FMCE Score	37% (s.d. = 19%)	35% (s.d. = 22%)
Phys 1 FMCE Score (excluding Phys 1A students)	61% (s.d. = 26%)	56% (s.d. = 27%)
Phys 2A CSEM Score	36% (s.d. = 9.5%)	27% (s.d. = 12%)
Phys 2 CSEM Score (excluding Phys 2A students)	48% (s.d. = 21%)	43% (s.d. = 19%)

One limitation to this analysis is that the selection of students to participate in Phys 1A and 2A was not random. Students were recruited into Phys 1A based on FMCE scores. We were able to control for differences in students’ academic preparation, but not other student characteristics, such as growth mindset, test anxiety, and other social psychological factors. However, in our previous analysis of performance in Phys 1 at Stanford, we found no impact of various social-psychological factors (including test anxiety) on final exam grades after controlling for academic preparation. Thus, it seems likely that the analysis we have conducted is a fair comparison for students who did and did not enroll in Phys 1A or 2A.

### Differences between 2017 and 2018 in Phys 2A

As noted, Phys 2A was much more effective in 2017 than in 2018, and only in 2017 was it providing the greatest benefit to the less prepared students (Table 4). Here we explore possible explanations for these differences.

Almost all the students who took Phys 2A in 2017 also took Phys 1A, while only half of the 2018 Phys 2A students took Phys 1A. So, one potential explanation for the 2017–18 difference is that Phys 2A was only effective when paired with Phys 1A. To test this explanation, we ran the 2018 model from Table 4 including an interaction term between Phys 1A and Phys 2A. This showed that the benefits of taking Phys 2A were the same, whether or not a student took Phys 1A, indicating this was not the explanation for the difference.

A second potential explanation is a slight shift in course structures between 2017 and 2018. In 2018, students stopped attending the regular Phys 2 discussion sections and instead attended a special section just for Phys 2A students (along with the regular Phys 2A course). This was done because of consistent feedback from students in 2017 that they were completely lost during Phys 2 discussion sections, as the other students and TAs rushed through the material too fast for them to follow. The Phys 2A special discussions focused on conceptual understanding of E&M. It seems unlikely that this shift would make Phys 2A *less* effective, as students likely learned more from these special discussion sections than they would in regular discussion sections, which were unstructured and attended mainly for attendance points. Note that, in both years, students spent the same total amount of time in class.

A more plausible explanation for at least some of the 2017–2018 difference concerns the teaching. In 2017, both instructors of Phys 2A were highly experienced at teaching in active learning settings. In 2018, one of the instructors was a new teacher, and thus may not have been as effective. However, both instructors used the same materials and class structure, and the new teacher had previously been a TA for Phys 2A, so it seems unlikely the difference in teacher effectiveness would be very large.

The explanation that we find most likely is the change in the student population. The recruitment process for Phys 2A changed between 2017 and 2018. In 2017, Phys 2A was open to any student who wished to take it, and there was only one section offered. In 2018, there was an application process to enroll in the course and recruitment that explicitly encouraged the less prepared students to enroll and discouraged better prepared students, and there were two sections available. As a result, there were twice as many students in 2018, and they were less prepared. The CSEM pre-scores were 0.33 standard deviations higher in 2017 compared with 2018, and the SAT/ACT math scores were 0.63 standard deviations higher in 2017. Thus, in 2018, the preparation gap between the Phys 2 students in Phys 2A, and those not in 2A was larger, and the same SI had a smaller impact. This is consistent with the explanation that we proposed above for the difference in effectiveness between Phys 1A and Phys 2A: if the gap in preparation between students enrolled in SI and the ones who are not enrolled is too large, there will be little benefit for a modest two-hour SI instruction to be effective.

This explanation appears to be at odds with the significant interaction term in Table 4, -0.36* (0.18), indicating that Phys 2A selectively benefitted the less well-prepared students in 2017. How can an intervention simultaneously benefit this population, but not be effective if the students are too far behind? We hypothesize that it has to do with the average preparation level in the SI course. If some students in the SI course have moderate levels of preparation, they can help the less prepared students by serving as peer “instructors”. However, if all students are quite poorly prepared, then the students might experience scenarios in which no one in the group knows what to do, so little progress is made [31]. This would suggest that Phys 2A was less effective in 2018 because the average level of preparation in the course was lower, and thus there were no sufficiently well prepared students to help the students with the lowest levels of preparation. Similarly, in Phys 1A, the average preparation level was too low, and there were no better prepared students to help lift up the students with lower levels of preparation. One can think of this in terms of Vygotsky’s “zone of proximal development”. The size of that zone depends both on the preparation of individual students and the range of preparation across the group. If the preparation level of the individual is high enough, they can benefit from the range of preparation of the group, with larger being better. However, if the preparation of the individual student is too low, or the range of preparation of the group is too low, a student will not benefit as much. All students in Phys 1A and quite poorly prepared relative to the average Phys 1 student, and the intervention was simply insufficient to help them catch up. We hypothesize that this was also the case for a greater fraction of Phys 2A students in 2018 than was the case in 2017.

### Comparison with previous results

Our finding that Phys 2A is effective is in line with previous results suggesting that SI is an effective strategy in physics [11,12]. Unlike the previous studies that report results from physics, our study uses measures of students’ prior physics and mathematics preparation to control for potential population differences in SI courses that lead to self-selection bias in reported results. Indeed, our findings for Phys 2A agree with the findings of Stanich et al., which was a randomized controlled study [9], though the magnitude of the effects we find in this study are somewhat larger. Indeed, Dawson et al. [13] also find other studies suggesting that SI is effective after controlling for student’s prior academic achievement.

To our knowledge, there are no published studies that align with our findings that Phys 1A is *not* effective. Null results are rarely published in educational research, so the lack of prior studies reporting this is not surprising. Additionally, we are not aware of any universities that have done rigorous internal reviews of their own SI programs and similarly found no positive effects. We hope that with this study, we will encourage more institutions to critically examine their SI programs so that we can assemble a more comprehensive feature of what makes SI work or not.

## Conclusions

We present a mixed result for the effectiveness of a cooperative group problem-solving based SI course. In the cases presented here, with a very similar instructional team and an identical instructional approach, the results vary drastically across different courses. In 2017, the group problem-solving model worked well for the introductory E&M course and improved all students’ performance, but that improvement was more pronounced for the less prepared students. In 2018, we found that the intervention benefitted all students equally, but the effect size was about half as large. However, the SI course did not improve the performance of students in the introductory mechanics course. The results presented here indicate that designing an effective SI course is complex and demands careful examination of the course and the student population. We were able to test and rule out many potential explanations for these varying results. The one explanation that we find the most plausible was that the SI course was not effective if the students enrolled were too far behind the other students in the target course. We are unable to provide data to confirm this explanation, but we can argue that would be true in the limiting cases. If freshman students are placed in an advanced graduate course, a modest supplemental instruction will make no difference, and if all students in a course are completely equivalent, giving a subset of them two hours of additional well-designed instructional time is almost certain to make a measurable difference.

We conclude that a modest amount of additional instructional time does not necessarily translate to better student outcomes, even with good teaching methods. We hypothesize that this is especially true when the preparation gap to be bridged is large, as was the case here in Phys 1, though do not have enough data to prove this hypothesis. Although this work is only looking at two courses at one institution over two years, we think it is an important example that raises questions about the underlying assumption behind supplemental instruction, namely that more well-designed instruction time translates to better student outcomes. As institutions and instructors seek to help their students with relatively weak high school preparation to succeed, future work should carefully measure the impact of the supplemental instruction they provide. Also, instructors and researchers need to further examine the complexities of designing effective supplemental instruction for different courses and student populations. It is likely that when the differences in preparation are too large, they are better addressed by having additional courses or some other instructional interventions, rather than supplemental instruction in existing courses.

## Supporting information

S1 File (CSV)

S2 File (CSV)

S3 File (CSV)

S4 File (CSV)
